# The significance of m6A RNA methylation modification in prognosis and tumor microenvironment immune infiltration of cervical cancer

**DOI:** 10.1097/MD.0000000000029818

**Published:** 2022-06-30

**Authors:** Yilin Guo, Yangyang Bai, Lu Wang, Zhen Xu, Xiliang Wang, Wuliang Wang

**Affiliations:** a Department of Gynecology and Obstetrics, The Second Affiliated Hospital of Zhengzhou University, China; b Henan Gynecological diseases (Gynecology Oncology) Clinical Research Center, Zhengzhou, China; c Department of Urology, Henan Provincial Hospital of Traditional Chinese Medicine, Zhengzhou, China.

**Keywords:** cervical cancer (CC), immunotherapy, N6-methyladenosine (m6A), prognosis, tumor microenvironment (TME)

## Abstract

Recent studies have highlighted that N6-methyladenosine (m6A) plays a significant role in tumorigenicity and progression. However, the mechanism of m6A modifications in the tumor microenvironment (TME) immune cell infiltration in cervical cancer (CC) remains unclear.

Clinical and RNA sequencing data of 25 m6A RNA methylation regulators were acquired from the Cancer Genome Atlas (TCGA) and Gene Expression Omnibus (GEO) database. LASSO Cox regression analysis was used to generate a prognostic risk signature. m6A modification patterns were identified based on the expression of 25 m6A regulators, and their correlation with TME immune cell-infiltrating characterization was analyzed. Principal component analysis was used to construct an m6A-scoring signature (m6A score) to evaluate the m6A modification patterns of individual CC samples and guide the selection of more effective immunotherapeutic strategies.

Genetic and expression alterations of 25 m6A regulators were highly heterogeneous between CC and normal tissues. METTL14 and IGF2BP1 were selected to conduct the prognostic risk signature. Three m6A modification patterns were identified in 659 CC samples, which were associated with distinct clinical outcomes and biological pathways. The TME immune cell-infiltrating characterization of the three m6A modification patterns was highly consistent with 3 tumor immune phenotypes, including immune-excluded, immune-inflamed, and immune-desert phenotypes. Due to the heterogeneity of m6A modification patterns, an m6A scoring signature was established to evaluate the m6A modification patterns of individual CC samples. Univariate and multivariate Cox regression analyses revealed that the m6A score is a robust and independent prognostic biomarker for assessing the prognosis of CC patients. A low m6A score, characterized by higher somatic mutation and higher expression of proliferation-related and DNA repair-related genes, indicated poor overall survival. Activation of immune infiltration was exhibited by the high m6A score, which was likely to have a good response and clinical benefits to antiPD-1/L1 immunotherapy.

This study highlights the prognostic value of 25 m6A regulators in CC. The m6A modification is related to immune regulation and the formation of TME heterogeneity and complexity. An m6A scoring signature to clarify the individual m6A modification pattern could enhance our understanding of TME immune cell-infiltrating characterization and guide immunotherapy strategies.

## 1. Introduction

Cervical cancer (CC) is the fourth leading cause of cancer-associated mortality in women worldwide, seriously threatening women’s physical and mental health.^[1]^ The predominant cause of CC is persistent infection with human papillomavirus (HPV).^[2]^ HPV vaccines, together with a growing arsenal of HPV-based screening tests, can dramatically decrease the risk of dying from CC.^[3,4]^ Currently, surgical removal of cervical tumors through radical hysterectomy is the treatment of choice for early stage CC, and concurrent chemoradiation is the preferred modality for the treatment of locally advanced CC.^[5]^ Early stage CC has an excellent long-term prognosis, and survival decreases markedly for both locally advanced and metastatic disease; both are associated with a higher risk of recurrence. Few effective treatment options exist for persistent, recurrent, or metastatic CCs.^[6]^ Therefore, the identification of novel targets and prognostic biomarkers for CC is of profound significance.

N6-methyladenosine (m6A) is regarded as the most important and abundant mRNA modification in eukaryotes.^[7]^ m6A methylation tends to occur in an RRACH (*R* = A or G, H = A, C, or U) consensus motif near the stop codon and 3’ untranslated terminal region of mRNA.^[8,9]^ The modification of m6A is catalyzed by 3 different m6A regulators, including m6A methyltransferases (METTL3, METTL5, METTL14, METTL16, WTAP, ZC3H13, RBM15, and RBM15B, termed as “writers”), demethylases (FTO, ALKBH3, and ALKBH5, termed as “erasers”), and m6A-binding proteins (YTHDC1, YTHDC2, YTHDF1, YTHDF2, YTHDF3, HNRNPC, FMR1, LRPPRC, HNRNPA2B1, IGF2BP1, IGF2BP2, IGF2BP3, RBMX, and ELAVL1, termed as “readers”).^[10]^ m6A regulators are implicated in various biological processes, including proliferation, invasion, and metastasis.^[11]^ However, the role of m6A regulators in the occurrence and prognosis of CC remains unclear.

Tumor microenvironment (TME) plays a crucial role in tumorigenesis, metastasis, and therapeutic efficacy.^[12]^ In the TME, tumor-infiltrating lymphocytes, including T lymphocytes, B lymphocytes, and antigen-presenting dendritic cells, mediate immunosuppression, which can help tumor cells achieve immune escape.^[13,14]^ Therefore, different tumor immune phenotypes may be identified by parsing TME landscape heterogeneity and complexity, and the ability to accurately predict the clinical efficacy of different immunotherapeutic approaches would also be improved.^[15]^ Recently, a study revealed not only a special correlation between TME infiltrating immune cells and m6A modification, but also predicted immunotherapeutic responsiveness and prognosis.^[16]^ Here, the expression patterns and prognostic value of m6A regulators in CC were systematically assessed through extensive bioinformatics analyses. An m6A-scoring signature was constructed to evaluate the m6A modification patterns of individual CC samples and to guide the selection of more effective immunotherapeutic strategies.

## 2. Methods

### 2.1. Datasets’ acquisition

The Cancer Genome Atlas (TCGA)-Cervical Squamous Cell Carcinoma and Endocervical Adenocarcinoma (CESC), a dataset that included RNA sequencing data, genome mutation data, and clinical data was downloaded from TCGA (http://cancergenome.nih.gov/, accessed on November 29, 2021). GSE52903 and GSE44001 (datasets that included RNA sequencing data and clinical data) were downloaded from the Gene Expression Omnibus (GEO) (https://www.ncbi.nlm.nih.gov/geo/, accessed on November 29, 2021). The transcripts per kilobase million (TPM) values were closer to the data of the GEO chip. For the TCGA-CESC dataset, the “limma” package in *R* was used to convert the fragments per kilobase of transcript per million (FPKM) value of the RNA data to the TPM value. The converted TCGA-CESC dataset was merged with the GSE52903 and GSE44001 datasets, and the merged data were corrected by “Combat” function in “sva” package. Principal component analysis (PCA) was used to determine correction results.

### 2.2. Selection and differential expression analysis of m6A RNA methylation regulators

We searched the literature for reports related to m6A methylation regulators, and finally selected 25 m6A regulators, including 8 writers (METTL3, METTL5, METL14, METL16, WTAP, ZC3H13, RBM15, and RBM15B), 14 readers (YTHDC1, YTHDC2, YTHDF1, YTHDF2, YTHDF3, HNRNPC, FMR1, LRPPRC, HNRNPA2B1, IGF2BP1, IGF2BP2, IGF2BP3, ELAVL1, and RBMX), and 3 erasers (FTO, ALKBH3, and ALKBH5). The “limma” package in *R* was used to identify differential expression of the m6A regulators between the CC samples and normal cervical samples. Gene expression levels, as well as the correlation with clinicopathological features, were visualized by heatmaps drawn with “pheatmap” package. The “corrplot” package was employed to reveal the correlation among m6A RNA regulators.

### 2.3. Construction of the prognostic signature

To evaluate the prognostic value of 25 m6A RNA regulators, we performed univariate Cox regression analyses of their expression to determine the prognostic value using the “Survival” package in *R* and plotted forest plots using the “forestplot” package. From this, it has been proven that 2 m6A regulators (METTL14 and IGF2BP1) were virtually associated with survival (*P* < .05), which were selected for further functional analysis and development of a potential risk signature in CC with the least absolute shrinkage and selection operator (LASSO) Cox regression algorithm. Then, METTL14 and IGF2BP1, and their coefficients were determined using the minimum criteria, choosing the best penalty parameter λ. The risk score was calculated as follows:


$$\text{Risk Score }=\;\sum_{i=1}^{N}coefi*xi$$

where N is the number of m6A regulators, Coefi is the coefficient, and x_i_ is the z-score transformed expression value of each selected regulator. This formula was used to calculate the risk score of each patient. Patients were divided into high-risk and low-risk subgroups based on the median risk scores of CC patient. The Kaplan–Meier survival curve was used to evaluate the survival differences between the groups, and the area under curve (AUC) value were used to assess the quality of the prognostic risk signature. Univariate and multivariate Cox regression analyses were conducted to compare the hazard ratios (HR) of prognostic signatures and important clinical features.

### 2.4. Experimental validation

To evaluate differences in METTL14 and IGF2BP1 expression at the protein level, Immunohistochemistry images of METTL14 and IGF2BP1 protein expression between CC and normal cervical tissues were downloaded by Human Protein Atlas (https://www.proteinatlas.org/) and analyzed. The mRNA expression of METTL14 and IGF2BP1 were quantified by quantitative real-time PCR (qRT-PCR). From January 2022 to March 2022, 10 CC tissues and paired normal cervical tissues were obtained from the Second Affiliated Hospital of Zhengzhou University. This study was approved by the Ethics Committee of the Second Affiliated Hospital of Zhengzhou University (2022131). All tissues were stored at −80 °C for RNA extraction. Total RNAs were extracted using the Trizol reagent (Invitrogen, Carlsbad, CA) according to the instructions of the manufacturer and treated with RQ1 DNase (Promega, Madison, WI) to remove DNA. The quality and quantity of the purified RNA were determined by measuring the absorbance at 260 nm and 280 nm (A260 and A280) using a SmartSpec Plus Spectrophotometer (Bio-Rad Laboratories, Inc., Hercules, CA). Reverse transcription reactions were carried out using ReverTra Ace qPCR RT Kit (TOYOBO Life Science, Shanghai, China). The Actin gene of human (species) was used as a control. Specific primers were designed based on cDNA sequences. Primer sequences were as follows:

(1) METTL14 5’-GAACACAGAGCTTAAATCCCCA-3’ (forward);

5’-TGTCAGCTAAACCTACATCCCTG-3’(reverse);

(2) IGF2BP1 5’-GCGGCCAGTTTCTTGGTCAA-3’ (forward);

5’-TTGGGCACCGAATGTTCAATC-3’(reverse);

(3) Actin 5’-TGGACTTCGAGCAAGAGATG-3’ (forward);

5’-GAAGGAAGGCTGGAAGAGTG-3’(reverse).

The qRT-PCR was performed on a Bio-Rad S1000 with Bestar SYBR GreenRT-PCR Master Mix (TOYOBO). PCR conditions consisted of denaturing at 95 °C for 1 minute, and 40 cycles of denaturing at 95 °C for 15 s followed by annealing and extension at 60 °C for 30 seconds. Relative gene expression was calculated using the 2^−ΔΔCt^ method normalized with the reference gene Actin.^[17]^ ΔCt was calculated by the value of Ct for METTL14 or IGF2BP1 minus the value of Ct for Actin, and ΔΔCt was calculated by the value of ΔCt for CC tissues minus the value of ΔCt for paired normal cervical tissues.

### 2.5. Unsupervised clustering for 25 m6A regulators

To investigate the various patterns of m6A modification based on the expression of 25 m6A regulators in CC, an unsupervised cluster analysis was conducted. The optimal number of clusters was selected according to the coefficients of dispersion, contour, and symbiosis. The “ConsensusClusterPlus” package in *R* was used to categorize patients with CC into 3 subgroups (50 iterations, resample rate of 80%).

### 2.6. Gene set variation analysis and gene enrichment function annotation

To investigate the different biological process of m6A modifications, the gene set variation analysis (GSVA) enrichment analysis was performed using “GSVA” packages in *R*. The gene sets of the “c2.cp.kegg.v7.4.symbols.gmt” were downloaded from the Molecular Signatures Database (MSigDB).^[18]^ Statistical significance was set at *P* < .05. The “clusterProfiler” package in *R* was conducted to perform the functional annotation of m6A regulators, with the cutoff value of false discovery rate < .05.

### 2.7. Analysis of immune cells infiltration based on single-sample gene set enrichment analysis

To quantify the infiltration levels of each immune cell types in the CC TME, Single-sample gene set enrichment analysis (ssGSEA) in *R* package “GSVA” was conducted. Most immune cell type-related marker genes were obtained from the study of Pornpimol Charoentong published by Bindea et al,^[19]^ which stored a variety of human immune cell subtypes including activated CD8^+^ T cells, activated dendritic cells, macrophages, and natural killer T cells. The relative abundance of each TME immune cell infiltration in CC patients was calculated using ssGSEA analysis.

### 2.8. Screening of differentially expressed genes between distinct phenotypes of m6A

Patients with CC were classified into 3 distinct m6A modification patterns based on previous unsupervised cluster analysis. The “limma” package in *R* was used to screen the differentially expressed genes (DEGs) between different modification patterns. An adjusted *P* value < .05 was considered as the significance criterion for determining DEGs. Similarly, univariate Cox regression analyses were conducted to screen for survival-related DEGs. The “ConsensusClusterPlus” package was used to identify distinct m6A gene patterns based on the survival-related DEGs (50 iterations, resample rate of 80%). Finally, the survival of distinct m6A gene patterns and differences in the expression of 25 m6A regulators were compared.

### 2.9. Generation of the m6A score

To identify the most suitable quantitative evaluation index of m6A modification patterns for individual CC patients, we constructed a set of scoring systems, termed the m6A score. The m6A score was generated by performing PCA using the formula:


$$\text{M6A Score }=\;\sum {PC1}_{i}+{PC2}_{i}$$

where PC1 represents principal component 1, PC2 represents principal component 2, and i represents survival-related DEGs. According to the correlation between m6A score and survival of the patients with CC, the “survminer” package in *R* was used to determine the cutoff point for the dataset subset, and the patients were divided into high m6A score subgroups and low m6A score subgroups based on the maximally selected rank statistics.

### 2.10. Statistical analysis

All statistical analyses and drawings were performed using *R* software (version 4.1.2). Wilcoxon test was used to compare the expression of m6A RNA methylation regulators between CC samples and normal cervical samples in the TCGA-CESC dataset. Compliant datasets were subjected to copy number variation (CNV) analysis. A plot of 25 m6A regulators CNV distribution in the chromosome was drawn using the “Rcircos” package in *R*. The 25 m6A regulators mutation data of CC was conducted using the “maftools” package. Spearman correlation was calculated for the correlation of different 25 m6A regulators and the relationship between the TME infiltrating immune cells and expression of m6A regulators. One-way ANOVA and Kruskal-Wallis tests were used as parametric and nonparametric methods, respectively, for the comparison of 3 or more groups. Kaplan-Meier and log-rank tests were performed to generate the survival curves of the prognostic analysis. Univariate and multivariate Cox regression analyses were used to compare the prognostic value of m6A scores and clinicopathological variables. All *P* values were 2-sided, with *P* < .05 defined as statistically significant.

## 3. Results

### 3.1. Landscape of genetic variation of m6A regulators in CC

**Figure 1.**
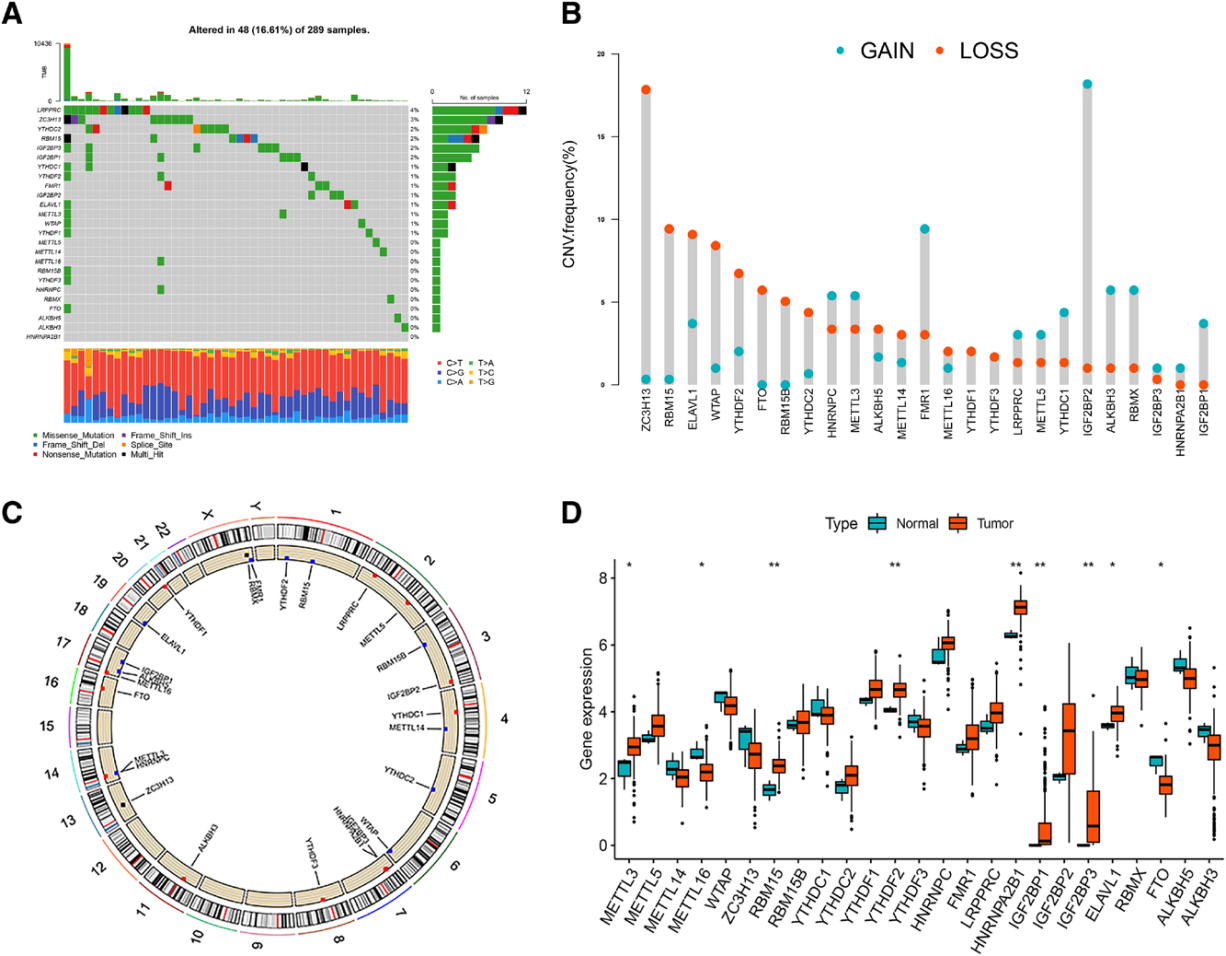
Landscape of genetic and expression variation of m6A regulators in cervical cancer. (A) The mutation frequency of 25 m6A regulators of cervical cancer patients in the TCGA-CESC cohort. (B) The CNV variation frequency of m6A regulators. Blue represents an increase in copy number, and red represents loss of copy number. (C) The location of CNV alteration of 25 m6A regulators. (D) The expression of 25 m6A regulators between normal tissues and tumor tissues. Red represents the tumor tissues, and blue represents the normal tissues. The asterisks represented the statistical *P* value (*P < .05; **P < .01; ***P < .001).

In this study, after duplicate samples from the same patients were excluded, a total of 304 CC samples and 3 normal cervical samples were enrolled for subsequent analysis. 25 m6A regulators, including 8 writers, 3 erasers, and 14 readers, were identified. First, we summarized the somatic mutations and CNV of 25 m6A regulators in the TCGA-CESC dataset. The results revealed that among 289 CC samples, only 48 samples (16.61%) experienced genetic alterations in the 25 m6A regulators. The highest mutation frequency of 4% was observed in LRPPRC, followed by ZC3H13. Three erasers (FTO, ALKBH3, and ALKBH5), 4 writers (METTL5, METTL14, METTL16, and RBM15B), and 4 readers (YTHDF3, HNRNPC, RBMX, and HNRNPA2B1) had no mutations detected, as shown in Figure 1A. Further analysis revealed that CNV alterations in the 25 m6A regulators were prevalent, with higher frequencies of CNV deletions in ZC3H13, RBM15, ELAVL1, and higher probabilities of CNV amplification in IGF2BP2, FMR1, ALKBH3, and RBMX (Fig. 1B). The positions of the CNV alterations in m6A regulators in chromosomes are shown in Figure 1C. Additionally, the expression of METTL3, RBM15, YTHDF2, HNRNPA2B1, ELAVL1, IGF2BP1, and IGF2BP3 in 304 CC samples was higher than that in 3 normal cervical samples. In contrast, the expression of METTL16 and FTO in normal cervical samples was higher than that in CC samples (Fig. 1D). Compared to normal cervical samples, m6A regulators with amplified CNV demonstrated markedly higher expression in CC samples (e.g., METTL3 and IGF2BP2), and vice versa (e.g., ZC3H13 and WTAP) (Fig. 1B, D). The above analyses presented the high heterogeneity of genetic and expressional alteration landscapes between CC and normal samples, and indicated that m6A regulators may be vital for CC occurrence and progression.

### 3.2. Correlation and prognostic value of m6A regulators in CC

**Figure 2.**
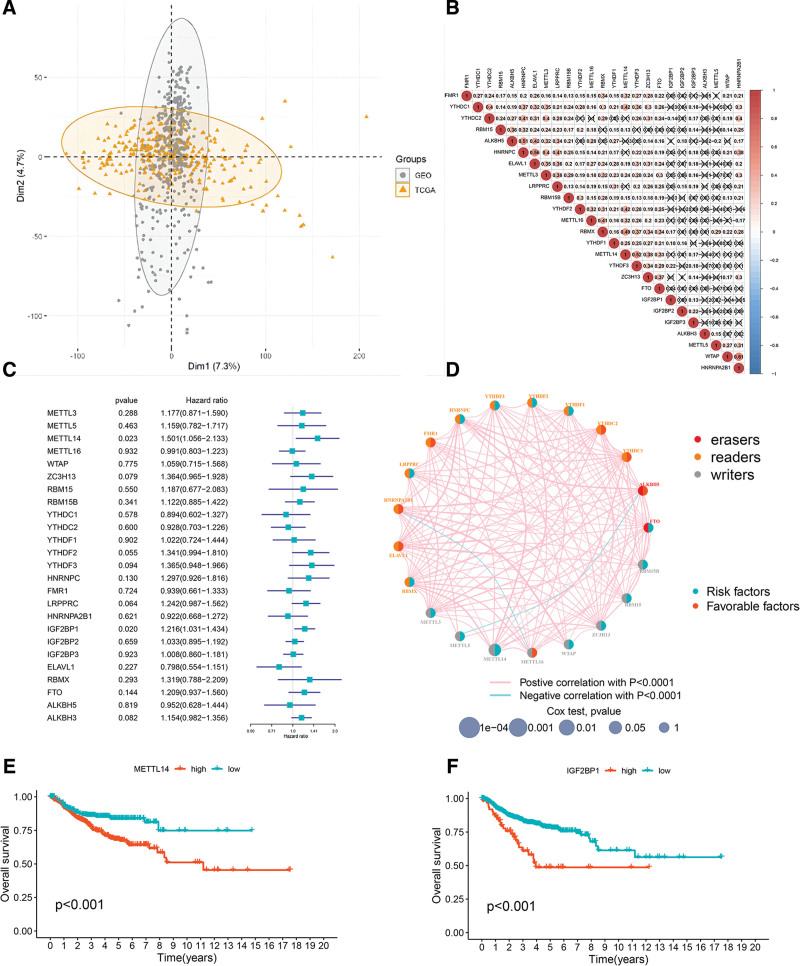
Correlation and prognostic value of m6A regulators in cervical cancer. (A) PCA analysis of TCGA-CESC datasets after transformation and 2 GEO datasets (GSE52903 and GSE44001). (B) Correlation analysis of m6A regulators expression in cervical cancer. Blue represents negative correlation, and red represents positive correlation. (D) Prognostic network of m6A regulators in cervical cancer. The circle size represented the effect of each regulator on the prognosis. Green dots in the circle, risk factors of prognosis; Red dots in the circle, protective factors of prognosis. The lines linking regulators showed their interactions, and thickness showed the correlation strength between regulators. Negative correlation was marked with blue and positive correlation with red. (C) Cox univariate analysis of the relationship between the expression level of each m6A RNA methylation regulators and the prognosis of cervical cancer. (E, F) Kaplan-Meier overall survival curves for METTL14 and IGF2BP1.

TCGA-CESC datasets after transformation were in consistency with 2 GEO datasets (GSE52903 and GSE44001) by PCA analysis (Fig. 2A) and the merged data, available survival and clinical information were collected for subsequent analyses. A total of 659 CC samples, including 55 samples in GSE52903, 300 samples in GSE44001, and 304 samples in TCGA-CESC datasets were enrolled in this study. Among 659 CC samples, median age at diagnosis was 49 (range 20–85 years) of which 67.8% were presented with stage I (Federation International of Gynecology and Obstetrics, FIGO 2014). Squamous cell carcinoma was the most prevalent histological type (79.7%) and 80.4% of CC samples were alive at the end of the follow-up (Table 1).

**Table 1:** Summary of the clinical characteristics of 659 CC samples.

Variable	TCGA-CESC (N = 304)	GSE52903 (N = 55)	GSE44001 (N = 300)	Total (N = 659)
Age				
>45	150 (49.3%)	33 (60.0%)	–	–
≤45	154 (50.7%)	22 (40.0%)	–	–
Median age	48 years	51 years	49 years	49 years
Stage (FIGO 2014)				
I	162 (53.3%)	27 (49.1%)	258 (86.0%)	447 (67.8%)
II	69 (22.7%)	8 (14.5%)	42 (14.0%)	119 (18.1%)
III	45 (14.8%)	16 (29.1%)	–	61 (9.2%)
IV	21 (6.9%)	4 (7.3%)	–	25 (3.8%)
Unknown	7 (2.3%)	–	–	7 (1.1%)
Histological type				
SCC	253 (83.2%)	51 (92.7%)	221 (73.7%)	525 (79.7%)
AC	47 (15.5%)	3 (5.5%)	64 (21.3%)	114 (17.3%)
ASC	4 (1.3%)	1 (1.8%)	15 (5.0%)	20 (3.0%)
Vital status				
Dead	70 (23.0%)	21 (38.2%)	38 (12.7%)	129 (19.6%)
Alive	234 (77.0%)	34 (61.8%)	262 (87.3%)	530 (80.4%)

AC = adenocarcinoma, ASC = adenosquamous carcinoma, FIGO = Federation of International of Gynecologists and Obstetricians, SCC = squamous cell carcinoma.

To further understand the interactions of 25 m6A regulators, we analyzed the correlations among these regulators in CC samples. A significant correlation was found among the 25 m6A regulators in the same category, as well as in erasers, readers, and writers. WTAP had the highest positive correlation with HNRNPA2B1 (correlation coefficient: .61), as shown in Figure 2B. Significant negative correlations between METTL5 and ALKBH5, as well as METTL16 and HNRNPA2B1, were observed (Fig. 2D).

Then, to investigate the prognostic value of the 25 m6A regulators in CC, we conducted univariate Cox regression analysis to calculate the hazard ratio (HR) for the m6A regulators (Fig. 2C). As a result, IGF2BP1 (*P* = .020, HR = 1.216, 95% CI HR 1.031–1.434) and METTL14 (*P* = .023, HR = 1.501, 95% CI HR 1.056–2.133) were considered risk factors. Kaplan-Meier survival curve analysis was performed to explore the prognostic significance of the 25 m6A regulators in CC. High expression levels of IGF2BP1, METTL14, METTL3, METTL5, FTO, ALKBH3, IGF2BP2, RBM15B, HNRNPC, YTHDF2, YTHDF3, and ZC3H13, as well as low levels of METTL16, HNRNPA2B1, YTHDC2, FMR1, and ELAVL1 were correlated with poor survival (*P* < .05), as shown in Figure 2E, 2F, and Supplementary Figure 1, Supplemental Digital Content, http://links.lww.com/MD/G836.

### 3.3. Construction of a prognostic risk signature of 2 m6A RNA methylation regulators

2 survival-associated m6A regulators, IGF2BP1 and METTL14 was selected to establish the prognostic risk signature to evaluate the ability of m6A regulators to predict the clinical outcomes of CC patients. Firstly, we collected 10 CC tissues and paired normal cervical tissues to verify the mRNA expression level of IGF2BP1 and METTL14. The median age of the ten patients was 52 years (range, 32–78) and 50.0% presented with stage I-II (FIGO 2014). The mRNA expression of IGF2BP1 and METTL14 was measured with qRT-PCR, and the results showed that the expression were significantly upregulated in CC tissues (Supplementary Fig. 2A, B, Supplemental Digital Content, http://links.lww.com/MD/G836). Immunohistochemical data from the Human Protein Atlas was used to evaluate the expression of IGF2BP1 and METTL14 at the protein level and the differential expressions were also confirmed, which was consistent with the results of the TCGA-CESC datasets and 2 GEO datasets (GSE52903 and GSE44001) (Supplementary Fig. 2C, D, Supplemental Digital Content, http://links.lww.com/MD/G836).

**Figure 3.**
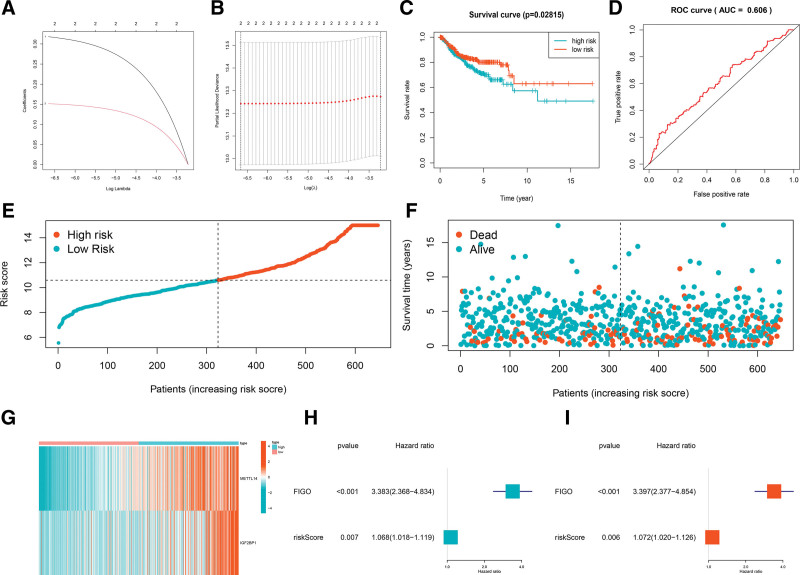
Construction of a prognostic risk signature of 2 m6A RNA methylation regulators. (A, B) The prognostic signature constructed by the minimum criterion of LASSO Cox regression algorithm. (C) Kaplan–Meier survival curve for high and low risk of cervical cancer patients; (D). ROC curve for 5-year survival of the risk prognostic signature. (E, F) Risk score and survival status for each cervical cancer patient. (H) The heatmap of the expression levels of 2 m6A regulators between high and low risk subgroups. (H, I) Univariate and multivariate Cox analysis of the clinicopathological features and risk prognostic signature.

Next, the LASSO Cox regression algorithm of IGF2BP1 and METTL14, was performed to establish a prognostic risk signature based on the minimum criteria (Fig. 3A, B). The coefficient of IGF2BP1 was .1515, and the coefficient of METTL14 was .3178. With the median risk score as the cutoff point, each CC patient was divided into high-risk and low-risk subgroups. There was a significant difference in the overall survival (OS) rate between the 2 subgroups, and the OS rate in the high-risk subgroup was significantly lower than that in the low-risk subgroup (*P* = .028, Fig. 3C). The ROC curve for 5-year survival illustrated the predictive performance of the prognostic risk signature (AUC = .606, Fig. 3D). The distribution of prognostic risk score, survival status, and the expression of 2 m6A regulators from each patient are also displayed (Fig. 3E–G). Univariate and multivariate Cox analyses were used to determine whether the prognostic risk signature was an independent predictor of CC. Univariate analysis showed that the FIGO stage (*P* < .001, HR = 3.383, 95% CI HR 2.368–4.834) and prognostic risk score (*P* = .007, HR = 1.068, 95% CI HR 1.018–1.119) were independent poor prognostic factors for CC patients (Fig. 3H). Multivariate analysis applying the same variables in the univariate analysis supported that FIGO stage (*P* < .001, HR = 3.397, 95%CI HR 2.377–4.854) and prognostic risk score (*P* = .006, HR = 1.072, 95%CI HR 1.020–1.126) were significantly associated with OS (Fig. 3I). All the above results showed that the 2 m6A regulators prognostic risk signature had a strong ability to predict the survival of CC patients.

### 3.4. m6A methylation modification patterns mediated by 25 m6A regulators

**Figure 4.**
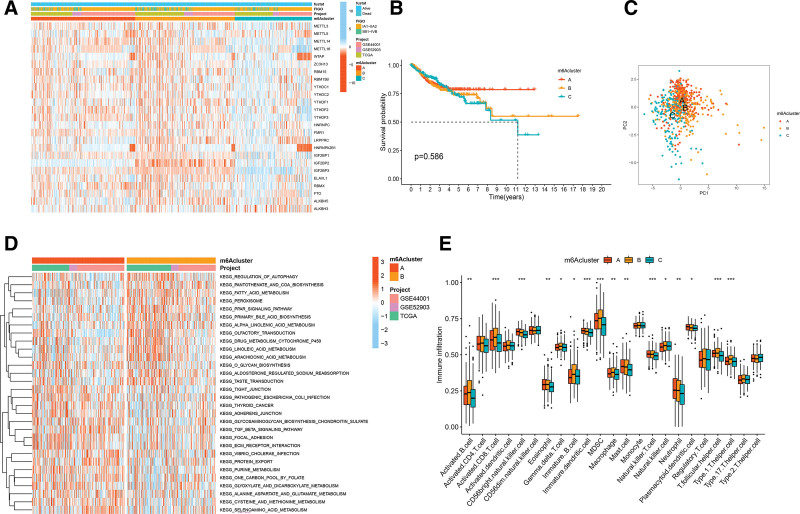
m6A methylation modification patterns mediated by 25 m6A regulators. (A) The heatmap of 3 m6A modification patterns by 25 m6A regulators. (B) Kaplan–Meier survival curve for 3 m6A modification patterns. (C) The scatter plot of PCA analysis. (D) GSVA analysis of functional pathways in Kyoto Encyclopedia of Genes and Genomes (KEGG) between m6Acluster A and m6Acluster B. (E) The abundance of each TME infiltrating cell in 3 m6A modification patterns. The asterisks represented the statistical *P* value (*P < .05; **P < .01; ***P < .001).

Based on the expression of 25 m6A regulators in CC, 3 distinct modification patterns were identified using unsupervised clustering (Supplementary Fig. 3A, B, Supplemental Digital Content, http://links.lww.com/MD/G836). These patterns are labeled as m6Acluster A-C, respectively (Supplementary Fig. 3C, Supplemental Digital Content, http://links.lww.com/MD/G836). The heatmap showed the highest expression of 25 m6A regulators in m6Acluster-B, while the lowest expression was observed in m6Acluster-C (Fig. 4A). Survival analysis of the 3 m6A modification patterns revealed a particularly prominent survival advantage in m6Acluster-A (Fig. 4B). The results of the PCA revealed significant differences in the transcriptome profiles of the 3 m6A modification patterns (Fig. 4C). To explore the biological functions of these distinct m6A modification patterns, we performed a GSVA enrichment analysis. We observed differences in functional pathways between different patterns, as shown in Figure 4D and Supplementary Figure 3D and 3E, http://links.lww.com/MD/G836. m6Acluster-A was markedly enriched in stromal and carcinogenic activation pathways such as extracellular matrix receptor interaction, TGF-β signaling pathway, focal adhesion, NOD-like receptor signaling pathway, and PPAR signaling pathway. m6Acluster-B presented enrichment pathways mainly concentrated in immune activation, including graft versus host disease, allograft rejection, complement and coagulation cascades, antigen processing and presentation, and cytokine-cytokine receptor interaction. m6Acluster-C was prominently related to cell cycle, mismatch repair, and apoptosis. Subsequent analyses of TME immune cell infiltration indicated that m6Acluster-B was remarkably rich in activated B cells, activated CD8^+^ T cells, myeloid-derived suppressor cells (MDSCs), macrophages, natural killer cells, and type 1 T helper cells, while m6Acluster-C was poor in TME immune cell infiltration (Fig. 4E). The above results showed that the 3 m6A modification patterns had significantly distinct TME cell-infiltrating characteristics. m6Acuster-A was characterized by stromal activation and activation of TGF-β signaling pathways, classified as an immune-excluded phenotype. m6Acuster-B was characterized by immune activation and immune cell infiltration, classified as an immune-inflamed phenotype. m6Acuster-C was characterized by the suppression of immunity, which is classified as an immune-desert phenotype.

### 3.5. m6A gene patterns mediated by m6A phenotype-related DEGs

**Figure 5.**
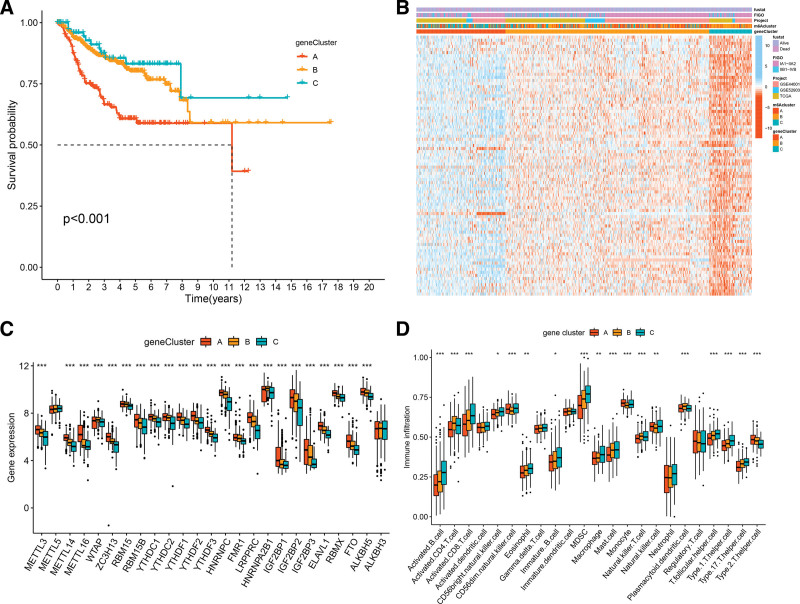
m6A gene patterns mediated by m6A phenotype-related DEGs. (A) Kaplan–Meier survival curve for 3 m6A gene patterns. (B) The heatmap of 3 m6A gene patterns. (C) The expression of 25 m6A regulators in 3 m6A gene patterns. The asterisks represented the statistical *P* value (*P < .05; **P < .01; ***P < .001). (D) The abundance of each TME infiltrating cell in m6A gene patterns. The asterisks represented the statistical *P* value (*P < .05; **P < .01; ***P < .001).

To detect the genetic features and potential biological behavior among the 3 m6A modification patterns in CC, we further identified 514 m6A phenotype-related DEGs (Supplementary Fig. 4A, Supplemental Digital Content, http://links.lww.com/MD/G836). The results of gene ontology (GO) enrichment analysis showed enrichment of biological processes related to methylation-dependent protein binding, methylated histone binding, regulation of mRNA metabolic process, and transcriptional coregulator activity (Supplementary Fig. 4B, Supplemental Digital Content, http://links.lww.com/MD/G836). To screen the DEGs related to prognosis, univariate Cox analysis was conducted and identified 84 DEGs that were significantly related to the prognosis of CC. To further validate this regulation mechanism, we performed an unsupervised cluster analysis based on 84 DEGs. Consistent with the m6A modification patterns, the clustering results revealed 3 m6A modification genomic phenotypes, and we termed these patterns as m6A gene clusters A–C, respectively (Supplementary Fig. 4C–E, Supplemental Digital Content, http://links.lww.com/MD/G836). Subsequent survival analysis revealed significant differences among the 3 m6A modification genomic phenotypes and the particularly prominent survival advantage in gene cluster C (*P* < .001, Fig. 5A). The heatmap shows the different clinicopathological characteristics of these patterns (Fig. 5B). The expression of 84 DEGs in gene cluster C was the highest, while that in gene cluster A was the lowest. 25 m6A regulators, except METTL5 and ALKBH3, were the source of prominent differences in the 3 m6A modification genomic phenotypes (Fig. 5C). In addition, to reveal the role of m6A modification of genomic phenotypes in TME immune regulation, we conducted ssGSEA analysis. The results indicated that immune cells in gene cluster C were more permeable, including activated B cells, activated CD8^+^ T cells, CD56bright natural killer cells, CD56dim natural killer cells, eosinophils, MDSCs, macrophages, mast cells, neutrophils, type 1 T helper cells, and type 17 T helper cells, while gene cluster A was poor in TME cell infiltration (Fig. 5D). Based on the above analyses, 3 distinct m6A methylation modification patterns were observed in the CC.

### 3.6. Generation and clinical correlation analysis of the m6A score in cervical cancer

**Figure 6.**
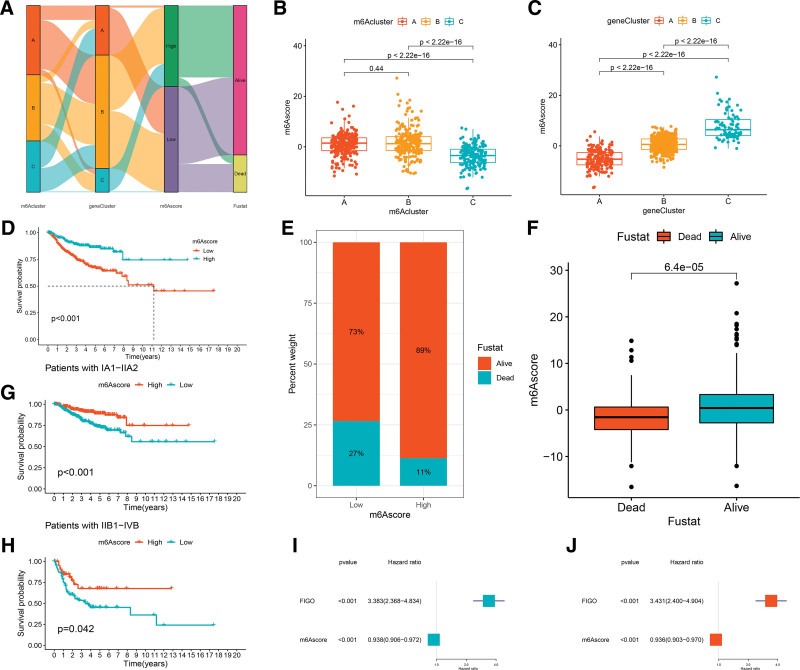
Generation and clinical correlation analysis of the m6A score in cervical cancer. (A) Alluvial diagram showing the association of m6A score groups with m6A clusters, gene clusters, and survival outcome. (B) Differences in m6Ascore among 3 m6A modification patterns. (C) Differences in m6Ascore among 3 m6A gene patterns. (D) Kaplan–Meier survival curve for high- and low-m6A score subgroups of cervical cancer patients. (E, F) Association of m6A score with survival status. (G, H) Stratified analysis of the m6A score for cervical cancer patients by FIGO state. (I, J) Univariate and multivariate Cox analysis of the clinicopathological features and m6A score.

The 3 m6A methylation modifications played a significant role in shaping distinct TME immune landscapes. However, these findings were largely based on the patient population, and the analysis could not be applied to accurately predict the patterns of m6A methylation modification in individual CC patients. Therefore, we established a scoring system to determine the m6A modification pattern in CC patients, named the m6A score. According to the optimal cutoff value, CC patients were divided into a high-m6A score subgroup and a low-m6A score subgroup. An alluvial diagram was constructed to visualize the m6A modification patterns of individual CC patients (Fig. 6A). There were significant differences in m6A scores between the m6Acluster (Fig. 6B). m6Acluster-C showed the lowest median score compared to the other clusters. Furthermore, differential expression analysis of m6A scores revealed significant differences in gene clusters (Fig. 6C). More importantly, gene cluster A had the lowest median score and gene cluster C presented the highest median score, which indicated that a high m6A score was related to immune-inflamed signatures, whereas a low m6A score was linked to immune-desert signatures.

Next, to further identify the prognostic value of m6A score, the survival analysis of the 2 m6A score subgroups revealed that CC patients with high m6A scores had a prominent survival benefit (*P* < .001, Fig. 6D). The mortality rate of CC patients in the low-m6A score subgroup (27%) was higher than that in the high-m6A score subgroup (11%, Fig. 6E, F). Stratified analysis indicated that the high-m6A score subgroup had a better prognosis than patients in the low-m6A score subgroup of FIGO state IA-IIA2 and FIGO state IIB1-IVB (Fig. 6G, H). In addition, we further evaluated whether the m6A score could serve as an independent prognostic biomarker of CC. Univariate and multifactorial Cox including FIGO status confirmed that m6A score was a robust and independent prognostic biomarker for assessing the prognosis of CC patients (Fig. 6I, J).

### 3.7. Characteristics of molecular subtype m6A and tumor somatic mutations

**Figure 7:**
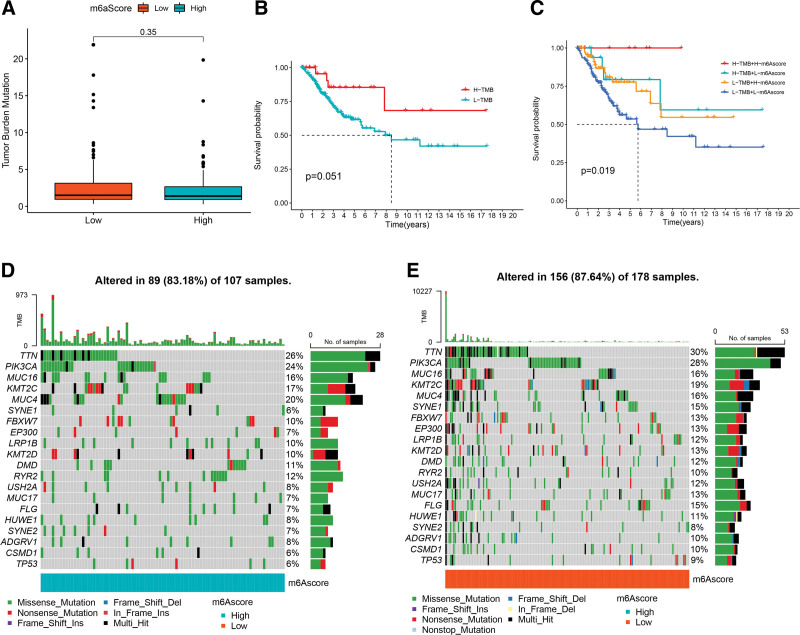
Characteristics of molecular subtype m6A and tumor somatic mutations. (A) Stratified analysis of the m6A score for cervical cancer patients by tumor mutation burden. (B) Kaplan–Meier survival curve for tumor mutation burden. (C) Kaplan–Meier survival curve for tumor mutation burden combined with m6A score. (D, E) The mutation frequency of high- and low-m6A score subgroups of cervical cancer patients.

Recent studies have revealed a significant correlation between somatic mutations in tumor genomes and immunotherapy response. Analysis of tumor mutation burden (TMB) showed no significant differences between the different m6A score subgroups (*P* = .35, Fig. 7A). According to the expression of TMB, CC patients were divided into a high-TMB subgroup and a low-TMB subgroup. Compared to the low-TMB subgroup, the high-TMB subgroup patients showed better survival results (Fig. 7B). More importantly, the survival curve of TMB combined with the m6A score showed that regardless of whether the m6A score was high or low, patients in the high-TMB subgroup consistently showed a significant survival benefit (*P* = .019, Fig. 7C). Furthermore, we analyzed the distribution differences of somatic mutations between the low-m6A score subgroup and high-m6A score subgroups in the TCGA-CESC dataset. As shown in Figures 7D and 7E, the low-m6A score subgroup presented a higher somatic mutation rate than the high-m6A score subgroup (87.64% vs 83.18%). The above results revealed a potential interaction between individual tumor somatic mutations and m6A methylation modification patterns.

### 3.8. Biological phenotypes and immune microenvironment of the m6A Score

The results of previous studies showed that a low m6A score in CC patients was associated with poor OS (*P* < .001, Fig. 6D). Therefore, to investigate the biological phenotypes related to the m6A score in CC, we compared the relationship between the m6A score and the expression levels of proliferation-related and DNA repair-related genes. Among proliferation-related genes, the low m6A score subgroup had the highest expression in *TP53*, *PCNA*, *JUN*, *NOTCH2*, *NOTCH3*, *MAPK3*, *MAPK8*, and *MAPK9*. (*P* < .05; Supplementary Fig. 5A, http://links.lww.com/MD/G836). Similarly, the expression levels of DNA repair-related genes *BRCA1*, *BRCA2*, *XRCC1*, *XRCC2*, *OGG1*, and *RRM1* also showed significant differences between the 2 m6A score subgroups (*P* < .05, Supplementary Fig. 5B, http://links.lww.com/MD/G836). These results suggest that with the upregulation of proliferation-related and DNA repair-related genes, a low m6A score is likely to be associated with poor prognosis.

**Figure 8.**
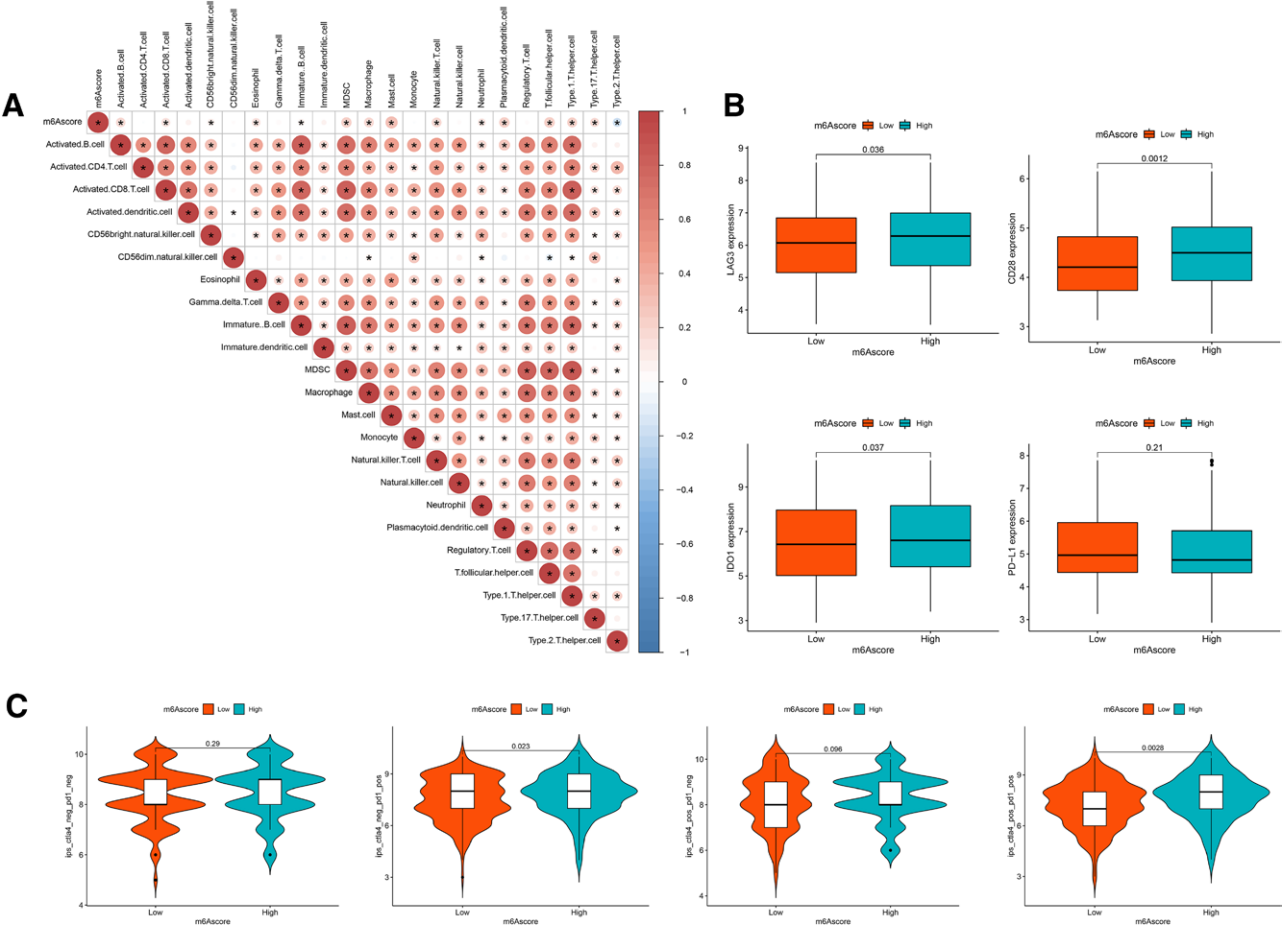
Biological phenotypes and immune microenvironment of the m6A Score. (A) The correlation between m6A score and TME immune cell infiltration. Blue represents negative correlation, and red represents positive correlation. (B) Expression level of immune checkpoints including Lymphocyte Activating 3 (LAG-3), CD28, Indoleamine 2,3-dioxygenase 1 (IDO1), programmed cell death-ligand 1 (PD-L1), between the 2 m6A score subgroups. (C) Immune response of high- and low-m6A score subgroups associated with CTLA-4 and PD-1.

Recently, immunotherapies represented by immune checkpoints, including PD-L1, PD-1, and CTLA-4, have undoubtedly emerged as a major breakthrough in cancer treatment. Unexpectedly, analysis of the correlation between the m6A score and TME immune cell infiltration revealed a significant association. Compared with the low-m6A score subgroup, the high-m6A score subgroup had higher levels of activated B cells, activated CD8^+^ T cells, CD56dim natural killer cells, MDSCs, macrophages, mast cells, natural killer T cells, neutrophils, type 1 T helper cells, and type 17 T helper cells, suggesting the characteristic of immune activation in high m6A score (Fig. 8A). Furthermore, we investigated the differences in the expression of immune checkpoints between the 2 m6A score subgroups. CC patients with high m6A scores showed obviously high expression of *LAG3*, *CD28*, and *IDO1* (Figure 8B). Next, the response to immune checkpoint inhibitor (ICI) treatment represented by CTLA-4/PD-1 inhibitors were further explored in immune cell proportion score (IPS) between the 2 m6A score subgroups. The results showed that CC patients in the high m6A score subgroup had higher IPS values of CTLA4 negative and PD-L1 positive (*P* = .023, Fig. 8C) and higher IPS values of CTLA4 positive and PD-L1 positive (*P* = .002, Fig. 8C) than the low-m6A score subgroups, which indicated that the high m6A score of CC patients presented a potential response to antiPD-1/L1 immunotherapy. Taken together, our findings strongly suggest that m6A methylation modification patterns, including the m6A score system we established, were significantly associated with tumor immune phenotypes, which could provide guidance for predicting the response to antiPD-1/L1 immunotherapy.

## 4. Discussion

CC is one of the most common gynecological malignant tumors worldwide, with approximately 604,127 new cases and 341,831 deaths annually in 2020.^[1,20]^ Notably, the incidence of CC is disproportionally distributed between developed and developing countries. With the development of organized screening and HPV vaccination programs, earliest stage CC patients have been detected promptly. However, locally advanced and recurrent CC lose the best treatment time and have few effective treatment options. m6A methylation is the most prevalent internal modification of RNA in eukaryotic cells and plays an indispensable role in the proliferation, invasion, and metastasis of cancer.^[21]^ However, to date, the potential role of m6A regulators in CC has not been comprehensively recognized. Therefore, identifying the role of distinct m6A modification patterns will enhance our understanding of the occurrence and prognosis of CC and guide more effective therapeutic strategies.

Here, we first analyzed the prognostic value of each 25 m6A RNA methylation regulators and developed a prognostic risk signature by applying 2 prognosis-associated m6A regulators, IGF2BP1 and METTL14, which the mRNA and protein expression of METTL14 and IGF2BP1 were verified in clinical CC tissues versus normal cervical tissues. The prognostic risk signature demonstrated good performance in predicting the survival outcome of CC and serves as a useful tool for predicting the prognosis of CC. Moreover, mRNA binding proteins of Insulin-like growth factor 2 (IGF2BP), including IGF2BP1, IGF2BP2, and IGF2BP3, serve as a distinct family of m6A readers. Recently, most of the cancer-related mRNA targets of IGF2BP1 have been shown to promote tumor proliferation.^[22]^ Huang et al^[23]^ demonstrated that IGF2BP could bind to the m6A sites of the *MYC* gene and loss of IGF2BP inhibited the proliferation and migration of hepatocellular carcinoma and CC. METTL14, an m6A methyltransferase, has been shown to be involved in tumorigenesis and development, whether as an oncogene or an antioncogene. It is expected that METTL14 could become a novel molecule for tumor therapy, but the comprehensive study of METTL14 in CC is still unclear.^[24]^

Complex and dynamic interactions exist between the tumor and the immune system, and different immune cell types play important roles in regulating tumor progression and development.^[25]^ Based on the expression of 25 m6A regulators in CC, we identified 3 distinct m6A methylation modification patterns, named m6Aclusters A-C. These 3 patterns showed significant differences in biological function and TME immune cell-infiltration characterization. m6Acluster-A was characterized by stromal activation and the activation of TGF-β signaling pathways, classified as immune-excluded phenotype; m6Acuster-B was characterized by immune activation and immune cell infiltration, classified as immune-inflamed phenotype; m6Acuster-C was characterized by the suppression of immunity, classified as immune-desert phenotype. The immune-inflamed phenotype, known as hot tumor, is characterized by the presence of massive immune cell infiltration in the TME and has a good immunotherapeutic effect, which results in a prominent survival advantage.^[26]^ The immune-desert phenotype, known as cold tumor, due to lack of activated and priming T-cells, is associated with immune tolerance and ignorance, and is insensitive to ICI therapy.^[27]^ Although the immune-excluded phenotype indicated the presence of massive immune cell infiltration, the immune cells were retained in the stroma surrounding the tumor cell nests rather than penetrating their parenchyma.^[28]^

Based on the above results of m6A modification patterns in CC, we further analyzed the mRNA transcriptome differences among the 3 patterns, and the DEGs were considered as m6A-related signature genes. Similar to the clustering results of the m6A modification patterns, 3 m6A modification genomic phenotypes, which serve as gene clusters A–C, were generated by unsupervised cluster analysis. Gene cluster C was also significantly correlated with stromal and immune activation. In addition, the prognosis of CC patients was significantly different among the 3 m6A modification genomic phenotypes. Therefore, a comprehensive assessment of m6A modification patterns will enhance our understanding of TME immune cell infiltration.

Considering the individual heterogeneity of m6A modification, there is an urgent need to quantify the m6A modification patterns of each CC patient. Interestingly, Zhu et al^[29]^ and Zhang et al^[30]^ constructed an m6A-scoring signature in bladder and gastric cancer, respectively, and demonstrated its prognostic value. In our study, we established a scoring system to determine the m6A modification pattern in CC patients, named the m6A score. Compared to patients with low m6A score, the high-m6A score of CC patients had a prominent survival benefit. Univariate and multivariate Cox regression analyses indicated that the m6A score is a robust and independent prognostic biomarker for assessing the prognosis of CC patients. In addition, we found that patients with low m6A scores had higher expressions of proliferation-related and DNA repair-related genes, and higher somatic mutation rates, suggesting an association with poor prognosis in patients with low m6A scores.

Recently, a new concept of the immune regulatory function of m6A regulators has been proposed.^[31]^ Wang et al^[32]^ reported that the loss of METTL3 or METTL14 increased cytotoxic tumor-infiltrating CD8^+^ T cells and elevated the secretion of IFN-γ, CXCL9, and CXCL910 in the TME in vivo. These findings suggest that METTL3 and METTL14 are potential therapeutic targets in anticancer immunotherapy Han et al^[33]^ reported that the loss of YTHDF1 in classical dendritic cells enhanced the cross-presentation of tumor antigens and the cross-priming of CD8+ T cells in vivo, and improved the therapeutic efficacy of PD-L1 checkpoint blockade. In this study, we found that the high m6A score was remarkably rich in activated B cells, activated CD8^+^ T cells, CD56dim natural killer cells, MDSCs, macrophages, mast cells, natural killer T cells, neutrophils, type 1 T helper cells, and type 17 T helper cells, while the low m6A score was poor in TME cell infiltration. Therefore, the m6A modification pattern characterized by an immune-inflamed phenotype exhibited a higher m6A score in CC. Previous studies revealed that the m6A scoring signature could guide immunotherapeutic strategies for gliomas, HCC, and pancreatic cancer.^[16,34,35]^ Our data also revealed that the m6A scoring signature was significantly associated with immune checkpoints. The high m6A score in CC patients is likely to have a good response to antiPD-1/L1 immunotherapy. Thus, it is suggested that the m6A score may be a reliable tool to evaluate the m6A modification patterns and TME immune cell-infiltrating characterization of individual CC patients and predict the clinical response to antiPD-1/PD-L1 immunotherapy.

This study has a few limitations that need to be acknowledged. First, we acquired 659 CC samples from the TCGA and GEO databases, while the clinical information of some samples, including age, TMN stage, and treatment potions, were incomplete, which could not be used to analyze the clinical value of the m6A scoring signature. Next, only 25 m6A methylation regulators were selected to explore the molecular mechanism of m6A modification, and no other regulators were incorporated. Finally, all data were extracted from online databases, and data from biochemical experiments for validation are lacking, which will be conducted in future research.

## 5. Conclusions

In conclusion, this study systematically demonstrated the expression pattern and prognostic value of m6A regulators in CC and constructed an m6A prognostic risk signature to predict the survival of CC patients. The difference among the 3 distinct m6A modification patterns based on 25 m6A regulators was a factor that could not be ignored because of the heterogeneity and complexity of individual TME. Due to the heterogeneity of m6A modification patterns, an m6A scoring signature was constructed to enhance our understanding of TME cell-infiltrating characterization and to guide more effective immunotherapy strategies.

### Acknowledgment

We thank all the researchers involved in the consolidation and submission of the data from the TCGA and GEO database, which may provide convenience and possibility of tumors studies in a large cohort.

### Author contributions

Conceptualization: Yilin Guo, Yangyang Bai, Wuliang Wang.

Data curation: Xiliang Wang.

Formal analysis: Yilin Guo, Yangyang Bai, Lu Wang

Funding acquisition: Yilin Guo, Wuliang Wang.

Methodology: Zhen Xu, Xiliang Wang.

Project administration: Xiliang Wang, Wuliang Wang.

Resources: Yilin Guo, Yangyang Bai, Zhen Xu.

Software: Yangyang Bai, Lu Wang, Xiliang Wang.

Supervision: Yilin Guo, Wuliang Wang.

Validation: Lu Wang, Zhen Xu.

Writing-original draft: Yilin Guo, Yangyang Bai, Lu Wang, Zhen Xu.

## Supplementary Material


