# Supplementary material for: The significance of m6A RNA methylation modification in prognosis and tumor microenvironment immune infiltration of cervical cancer
Source: Medicine (Baltimore). 2022 Jun 30;101(26):e29818. doi: 10.1097/MD.0000000000029818 (PMC9239609; doi:10.1097/MD.0000000000029818)
Supplement: Supplementary file 1 [file medi-101-e29818-s001.pdf]

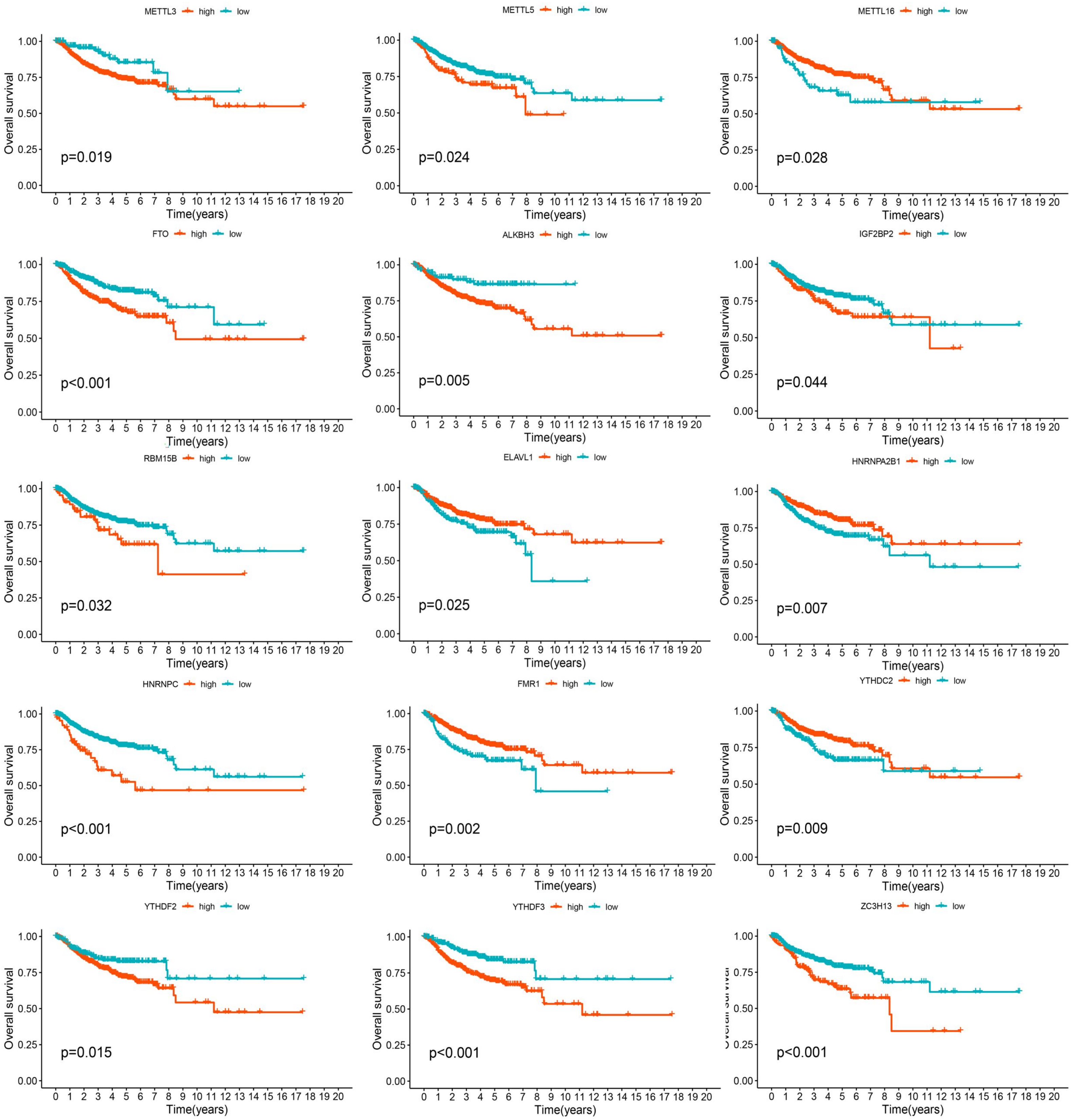
METTL3 high low
p=0.019
METTL5 high low
p=0.024
METTL16 high low
p=0.028
FTO high low
p<0.001
ALKBH3 high low
p=0.005
IGF2BP2 high low
p=0.044
RBM15B high low
p=0.032
ELAVL1 high low
p=0.025
HNRNPA2B1 high low
p=0.007
HNRNPC high low
p<0.001
FMR1 high low
p=0.002
YTHDC2 high low
p=0.009
YTHDF2 high low
p=0.015
YTHDF3 high low
p<0.001
ZC3H13 high low
p<0.001
Overall survival
Time(years)
0.00 0.25 0.50 0.75 1.00
0 1 2 3 4 5 6 7 8 9 10 11 12 13 14 15 16 17 18 19 20

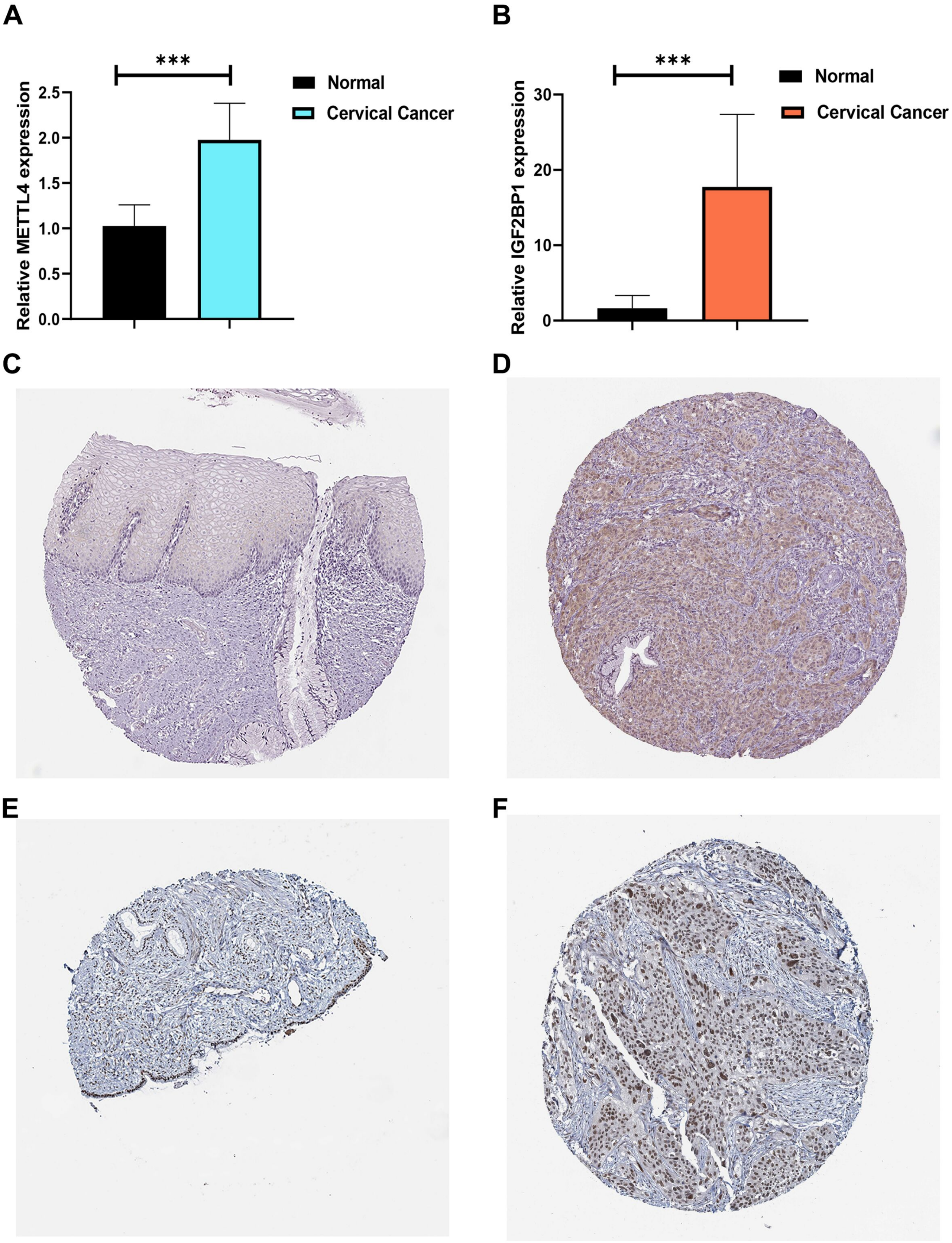
A
B
C
D
E
F
***
Normal
Cervical Cancer
Relative METTL4 expression
2.5
2.0
1.5
1.0
0.5
0.0
***
Normal
Cervical Cancer
Relative IGF2BP1 expression
30
20
10
0

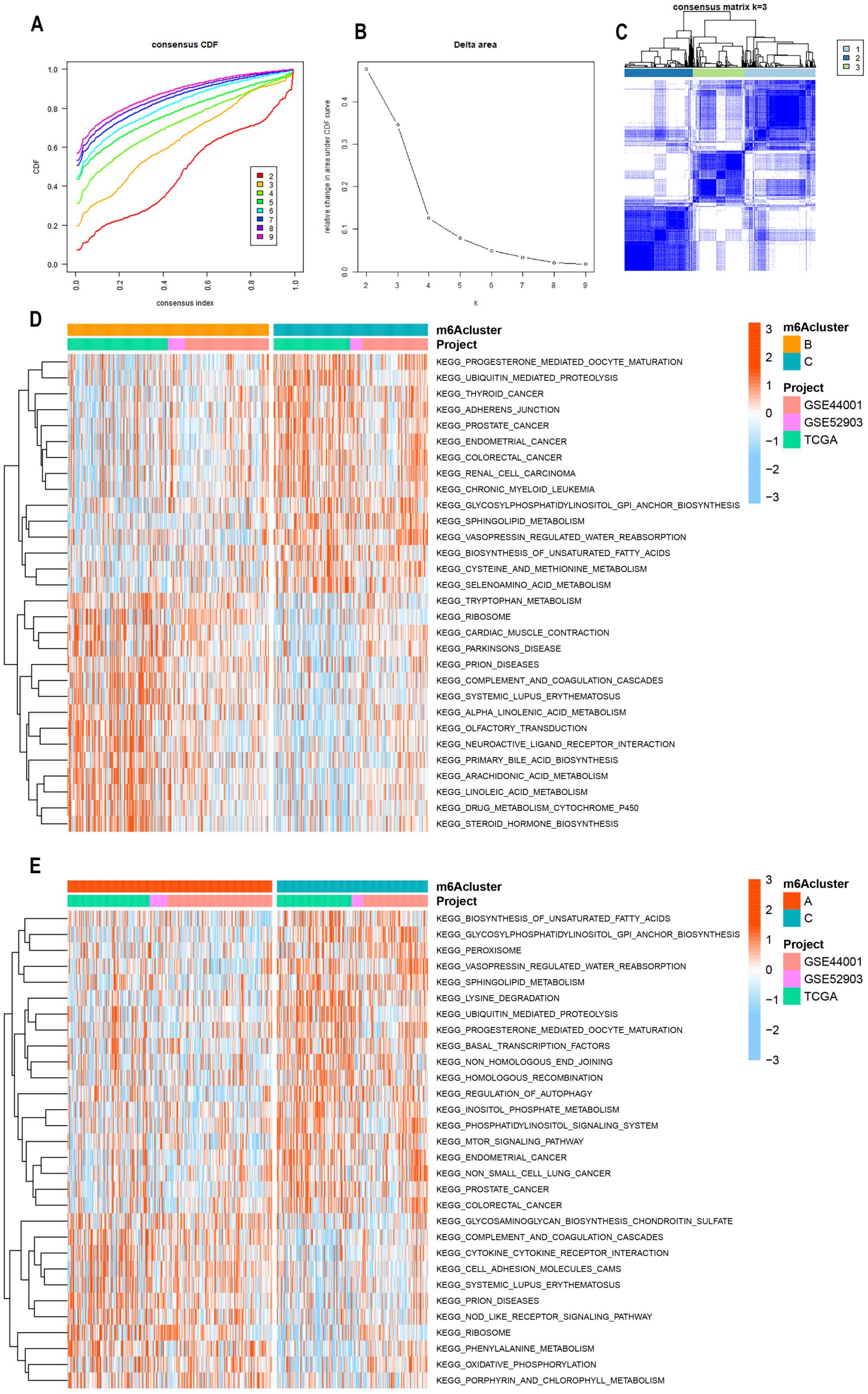
A
consensus CDF
CDF
consensus index
2
3
4
5
6
7
8
9
B
Delta area
relative change in area under CDF curve
k
C
consensus matrix k=3
1
2
3
D
m6Acluster
Project
B
C
GSE44001
GSE52903
TCGA
KEGG_PROGESTERONE_MEDIATED_OOCYTE_MATURATION
KEGG_UBIQUITIN_MEDIATED_PROTEOLYSIS
KEGG_THYROID_CANCER
KEGG_ADHERENS_JUNCTION
KEGG_PROSTATE_CANCER
KEGG_ENDOMETRIAL_CANCER
KEGG_COLORECTAL_CANCER
KEGG_RENAL_CELL_CARCINOMA
KEGG_CHRONIC_MYELOID_LEUKEMIA
KEGG_GLYCOSYLPHOSPHATIDYLINOSITOL_GPI_ANCHOR_BIOSYNTHESIS
KEGG_SPHINGOLIPID_METABOLISM
KEGG_VASOPRESSIN_REGULATED_WATER_REABSORPTION
KEGG_BIOSYNTHESIS_OF_UNSATURATED_FATTY_ACIDS
KEGG_CYSTEINE_AND_METHIONINE_METABOLISM
KEGG_SELENOAMINO_ACID_METABOLISM
KEGG_TRYPTOPHAN_METABOLISM
KEGG_RIBOSOME
KEGG_CARDIAC_MUSCLE_CONTRACTION
KEGG_PARKINSONS_DISEASE
KEGG_PRION_DISEASES
KEGG_COMPLEMENT_AND_COAGULATION_CASCADES
KEGG_SYSTEMIC_LUPUS_ERYTHEMATOSUS
KEGG_ALPHA_LINOLENIC_ACID_METABOLISM
KEGG_OLFACTORY_TRANSDUCTION
KEGG_NEUROACTIVE_LIGAND_RECEPTOR_INTERACTION
KEGG_PRIMARY_BILE_ACID_BIOSYNTHESIS
KEGG_ARACHIDONIC_ACID_METABOLISM
KEGG_LINOLEIC_ACID_METABOLISM
KEGG_DRUG_METABOLISM_CYTOCHROME_P450
KEGG_STEROID_HORMONE_BIOSYNTHESIS
E
m6Acluster
Project
A
C
GSE44001
GSE52903
TCGA
KEGG_BIOSYNTHESIS_OF_UNSATURATED_FATTY_ACIDS
KEGG_GLYCOSYLPHOSPHATIDYLINOSITOL_GPI_ANCHOR_BIOSYNTHESIS
KEGG_PEROXISOME
KEGG_VASOPRESSIN_REGULATED_WATER_REABSORPTION
KEGG_SPHINGOLIPID_METABOLISM
KEGG_LYSINE_DEGRADATION
KEGG_UBIQUITIN_MEDIATED_PROTEOLYSIS
KEGG_PROGESTERONE_MEDIATED_OOCYTE_MATURATION
KEGG_BASAL_TRANSCRIPTION_FACTORS
KEGG_NON_HOMOLOGOUS_END_JOINING
KEGG_HOMOLOGOUS_RECOMBINATION
KEGG_REGULATION_OF_AUTOPHAGY
KEGG_INOSITOL_PHOSPHATE_METABOLISM
KEGG_PHOSPHATIDYLINOSITOL_SIGNALING_SYSTEM
KEGG_MTOR_SIGNALING_PATHWAY
KEGG_ENDOMETRIAL_CANCER
KEGG_NON_SMALL_CELL_LUNG_CANCER
KEGG_PROSTATE_CANCER
KEGG_COLORECTAL_CANCER
KEGG_GLYCOSAMINOGLYCAN_BIOSYNTHESIS_CHONDROITIN_SULFATE
KEGG_COMPLEMENT_AND_COAGULATION_CASCADES
KEGG_CYTOKINE_CYTOKINE_RECEPTOR_INTERACTION
KEGG_CELL_ADHESION_MOLECULES_CAMS
KEGG_SYSTEMIC_LUPUS_ERYTHEMATOSUS
KEGG_PRION_DISEASES
KEGG_NOD_LIKE_RECEPTOR_SIGNALING_PATHWAY
KEGG_RIBOSOME
KEGG_PHENYLALANINE_METABOLISM
KEGG_OXIDATIVE_PHOSPHORYLATION
KEGG_PORPHYRIN_AND_CHLOROPHYLL_METABOLISM

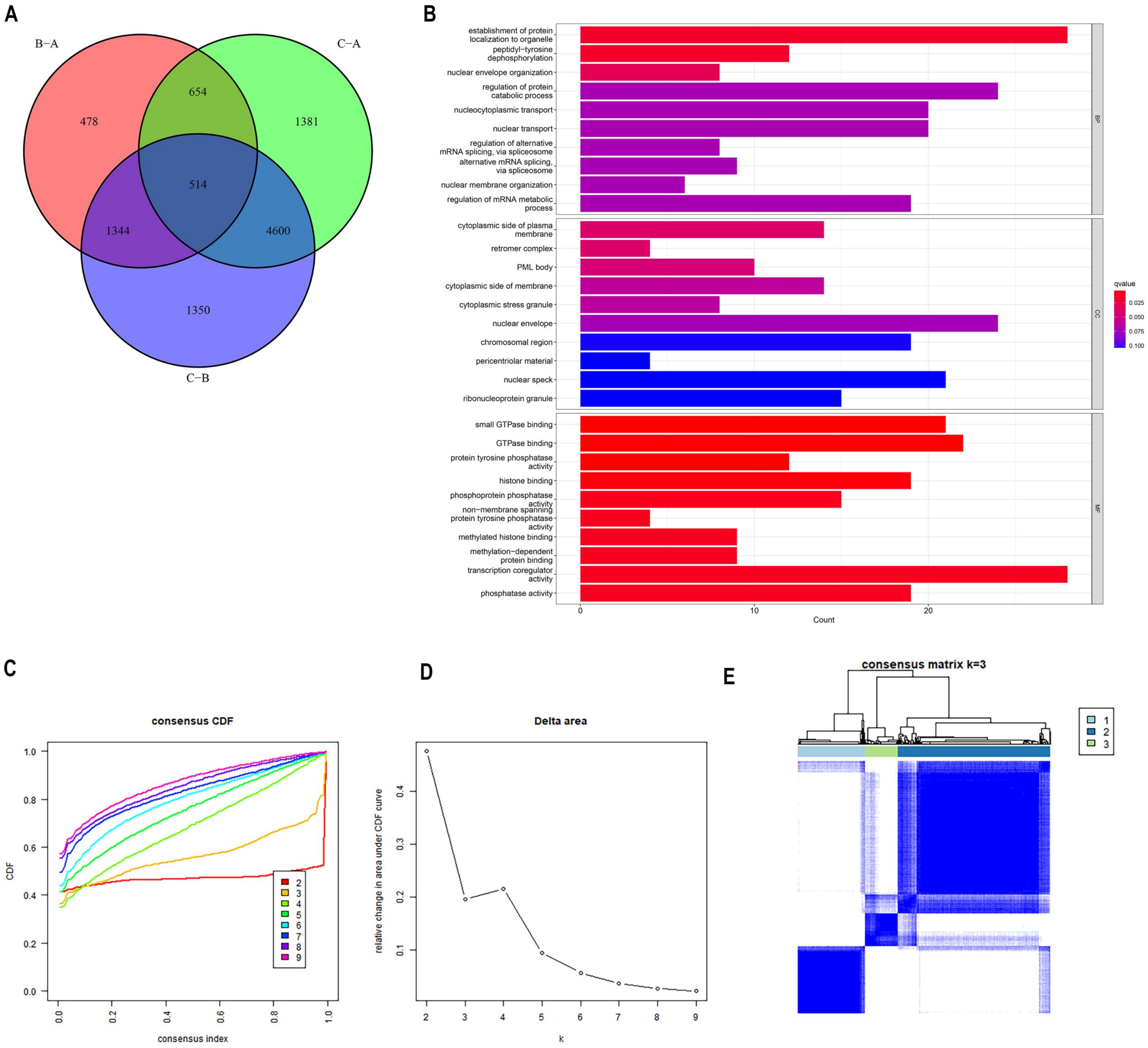
A
B−A
C−A
C−B
478
654
1381
514
1344
4600
1350
B
establishment of protein localization to organelle
peptidyl−tyrosine dephosphorylation
nuclear envelope organization
regulation of protein catabolic process
nucleocytoplasmic transport
nuclear transport
regulation of alternative mRNA splicing, via spliceosome
alternative mRNA splicing, via spliceosome
nuclear membrane organization
regulation of mRNA metabolic process
cytoplasmic side of plasma membrane
retromer complex
PML body
cytoplasmic side of membrane
cytoplasmic stress granule
nuclear envelope
chromosomal region
pericentriolar material
nuclear speck
ribonucleoprotein granule
small GTPase binding
GTPase binding
protein tyrosine phosphatase activity
histone binding
phosphoprotein phosphatase activity
non−membrane spanning protein tyrosine phosphatase activity
methylated histone binding
methylation−dependent protein binding
transcription coregulator activity
phosphatase activity
BP
CC
MF
qvalue
0.025
0.050
0.075
0.100
0
10
20
Count
C
consensus CDF
CDF
consensus index
0.0
0.2
0.4
0.6
0.8
1.0
2
3
4
5
6
7
8
9
D
Delta area
relative change in area under CDF curve
k
0.1
0.2
0.3
0.4
2
3
4
5
6
7
8
9
E
consensus matrix k=3
1
2
3

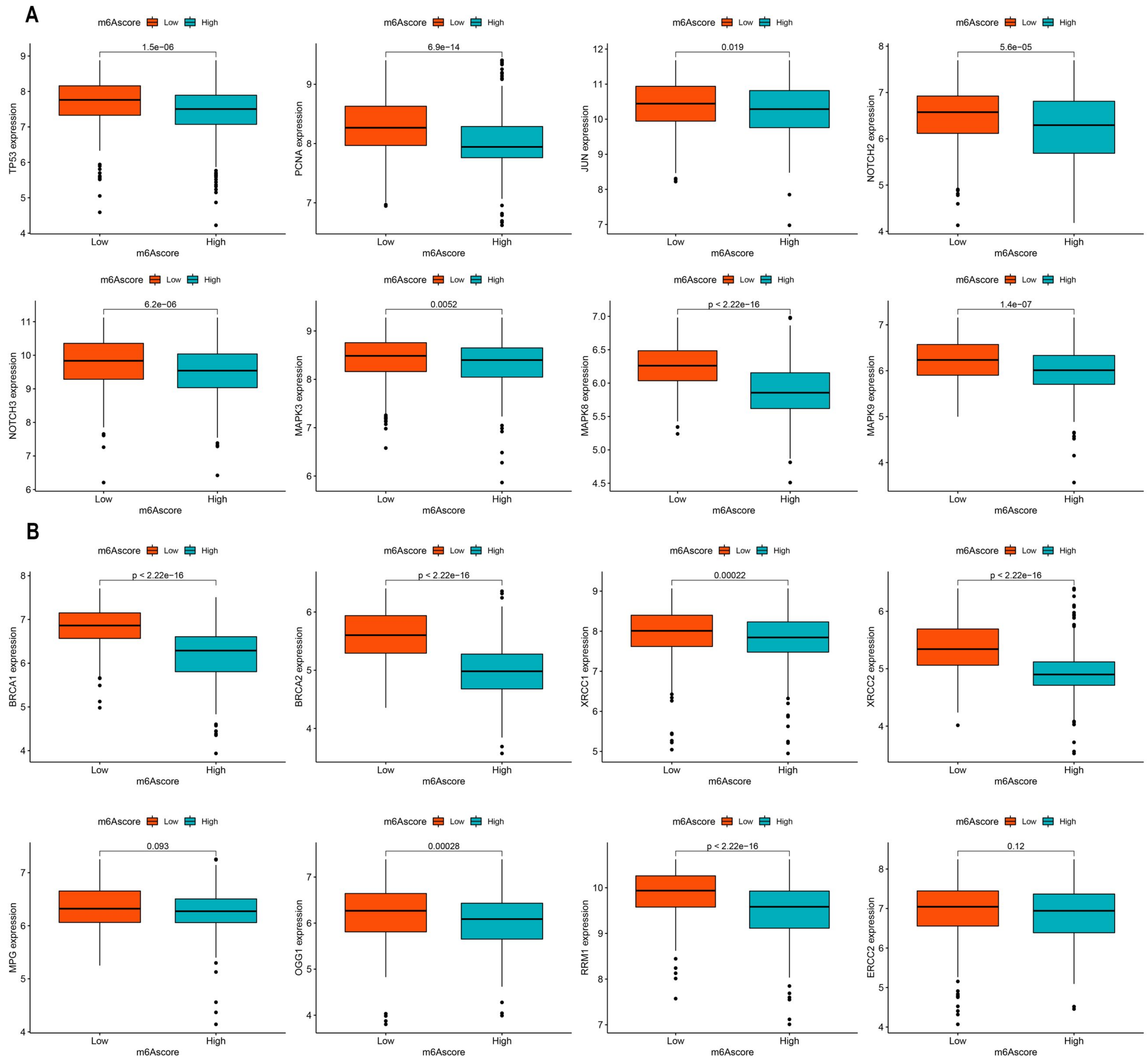

A
m6Ascore Low High
1.5e−06
TP53 expression
6.9e−14
PCNA expression
0.019
JUN expression
5.6e−05
NOTCH2 expression
6.2e−06
NOTCH3 expression
0.0052
MAPK3 expression
p < 2.22e−16
MAPK8 expression
1.4e−07
MAPK9 expression
B
p < 2.22e−16
BRCA1 expression
p < 2.22e−16
BRCA2 expression
0.00022
XRCC1 expression
p < 2.22e−16
XRCC2 expression
0.093
MPG expression
0.00028
OGG1 expression
p < 2.22e−16
RRM1 expression
0.12
ERCC2 expression
Low
High
m6Ascore

The significance of m6A RNA Methylation modification in prognosis and tumor microenvironment Immune infiltration of cervical cancer

Supplementary Figure Legend

Supplementary Figure 1 Kaplan–Meier survival curve for METTL3, METTL5, FTO, ALKBH3, IGF2BP2, RBM15B, HNRNPC, YTHDF2, YTHDF3, ZC3H13, METTL16, HNRNPA2B1, YTHDC2, FMR1, and ELAVL1.

Supplementary Figure 2 Experimental validation of METTL14 and IGF2BP1 performed by qRT-PCR and the Human Protein Atlas database. (A) The mRNA expression of METTL14 in Cervical cancer tissues versus normal cervical tissues. The asterisks represented the statistical p value (*$P < 0.05$; **$P < 0.01$; ***$P < 0.001$). (B) The mRNA expression of IGF2BP1 in Cervical cancer tissues versus normal cervical tissues. The asterisks represented the statistical p value (*$P < 0.05$; **$P < 0.01$; ***$P < 0.001$). (C) The protein expression of IGF2BP1 in normal cervical tissues (D) The protein expression of IGF2BP1 in cervical cancer tissues. (E) The protein expression of METTL14 in normal cervical tissues (F) The protein expression of METTL14 in cervical cancer tissues.

Supplementary Figure 3 Three m6A methylation modification patterns and the biological characteristics of each pattern. (A) Unsupervised clustering

cumulative distribution function (CDF) k=2–9. (B) Relative change in area under CDF curve for k=2–9. (C) Unsupervised clustering matrix for k=3. (D) GSVA analysis of functional pathways in Kyoto Encyclopedia of Genes (KEGG) between m6Acluster B and m6Acluster C. (E) GSVA analysis of functional pathways in Kyoto Encyclopedia of Genes (KEGG) between m6Acluster A and m6Acluster C.

Supplementary Figure 4 Unsupervised clustering of 84 prognostic differentially expressed genes (DEGs) between m6A. (A) Venn diagram of m6A phenotype-associated DEGs (B) Gene Ontology (GO) enrichment analysis of m6A phenotype-related DEGs. (C) Unsupervised clustering cumulative distribution function (CDF) k=2–9. (D) Relative change in area under CDF curve for k=2–9. (E) Unsupervised clustering matrix for k=3.

Supplementary Figure 5 Biological phenotypes of the m6A Score. (A) Expression levels of proliferation-related genes between the two m6A score subgroups. (B) Expression levels of DNA repair-related genes between the two m6A score subgroups.
